# Treatment of Internal Hæmorrhoids by Injections

**Published:** 1888-01

**Authors:** M. F. Clausius

**Affiliations:** Beecher, Illinois


### *TREATMENT OF INTERNAL HÆMORRHOIDS BY INJECTION.*

By M. F. CLAUSIUS, M. D., Beecher, Illinois.

In the *Medical Record* of June 25th, my attention was called to an article communicated by Dr. Shuford, of Tyler, Texas, regarding the treatment of internal hæmorrhoids by sub-mucous injection. To accomplish this, it is necessary to have an instrument that can be introduced with but little pain, and then so constructed as to expose as much of the mucous membrane as possible. Having introduced the speculum, the center of the hemorrhoidal tumor should be brought into plain view, and puncture the neec le at that spot, as it is less sensitive here than elsewhere.

For this procedure, a long needle is required; if possible it should bave a guard near its point so as not to enter too deeply. The following solution is recommended:

℞—Acid salicylas . . . . . . . . . . . . . . . .

Sodæ biboratis . . . . . . . . . . . . . . . . aa. ʒj.

Glycerinæ . . . . . . . . . . . . . . . . . . . ʒiij.

Acid carbol . . . . . . . . . . . . . . . . . . ʒij.

M. Sig.—Mix well and let stand for a short time, after which it will become perfectly clear. Of this solution, three to five drops are used for small tumors, and five to eight drops for larger ones.

This will cause them to become atrophied, and shrink up and peel off without pain, inflammation or suppuration. Each tumor ought to be injected separately. An interval of from eight to ten days should elapse between the injections, so that the mucous membrane becomes toughend.

The doctor reported many cases, all with good results. I happened to have, at the same time, a patient suffering from this trouble. I had used on him equal parts of glycerine and carbolic acid, and injected a few drops into the tumor, but this seemed to act rather slowly. I at once prepared the foregoing recommended formula, and used four drops for each tumor. I had to repeat this but twice, and the tumors have perfectly disappeared. The injections caused hardly any pain. The man, a farm laborer, could pursue his ordinary work, and has now fully recovered.

